# Bisphenol A Exposure and Asthma Development in School-Age Children: A Longitudinal Study

**DOI:** 10.1371/journal.pone.0111383

**Published:** 2014-10-30

**Authors:** Kyoung-Nam Kim, Jin Hee Kim, Ho-Jang Kwon, Soo-Jong Hong, Byoung-Ju Kim, So-Yeon Lee, Yun-Chul Hong, Sanghyuk Bae

**Affiliations:** 1 Department of Preventive Medicine, Seoul National University College of Medicine, Seoul, Korea; 2 Institute of Environmental Medicine, Medical Research Center, Seoul, Korea; 3 Department of Preventive Medicine and Public Health, Dankook University College of Medicine, Cheonan, Korea; 4 Department of Pediatrics, Childhood Asthma Atopy Center, Research Center for Standardization of Allergic Diseases, Asan Medical Center, University of Ulsan College of Medicine, Seoul, Korea; 5 Department of Pediatrics, Haeundae Paik Hospital, Inje University College of Medicine, Busan, Korea; 6 Department of Pediatrics, Hallym University Sacred Heart Hospital, Hallym University College of Medicine, Anyang, Korea; Institute for Health & the Environment, United States of America

## Abstract

**Background:**

Although the effect of bisphenol A on various health outcomes has been extensively examined, few studies have investigated its effect on asthma.

**Objective:**

We hypothesized that exposure to bisphenol A in school-age children was associated with wheezing and asthma.

**Methods:**

Participants included 127 children aged 7–8 years without a previous asthma diagnosis in an elementary school in Seoul, Korea. Three surveys were conducted, each 2 years apart. Bisphenol A concentration was measured at the baseline survey, and PC_20_, which is defined as the methacholine concentration that induces a decrease in FEV_1_ of 20% from baseline, was measured at every survey. Associations between bisphenol A concentration at 7–8 years of age and wheezing, asthma, and PC_20_ at ages up to 11–12 years were examined using generalized estimating equations, a marginal Cox regression model, and a linear mixed model.

**Results:**

The log-transformed creatinine-adjusted urinary bisphenol A concentration at 7–8 years was positively associated with wheezing (odds ratio, 2.48; 95% confidence interval, 1.15–5.31; *P* = .02) and asthma (hazard ratio, 2.13; 95% confidence interval, 1.51–3.00; *P*<.001) at ages up to 11–12 years. Bisphenol A was also negatively associated with PC_20_ (ß = −2.33; *P* = .02). When stratified by sex, the association between bisphenol A and asthma remained significant only in girls (hazard ratio, 2.45; 95% confidence interval, 2.18–2.76; *P*<.001).

**Conclusion:**

Increased urinary bisphenol A concentrations at 7–8 years old were positively associated with wheezing and asthma and negatively associated with PC_20_ at ages up to 11–12 years.

## Introduction

Asthma is one of the most common childhood diseases with prevalence of 9.1% in the United States and 7.6% in Korea [1]–[3]. The global prevalence of asthma in children has risen significantly over the recent decades [4], [5]. The exact cause of this increase is not known, but associations with increasing urbanization have been reported [6], [7]. Furthermore, the increase in global asthma prevalence has occurred within approximately the same timeframe as the widespread use of industrial chemicals like bisphenol A (BPA) [8]. BPA is one of the chemicals produced in the highest volumes worldwide [9] and is used in the production of polycarbonate plastics and epoxy resins. Polycarbonate plastics are used to make products such as water bottles, toys, dental sealants, and compact discs, whereas epoxy resins are used to coat the insides of cans for food and beverages [8], [10]. Human exposure to BPA is extensive, and 95% of the United States population has detectable urinary BPA concentrations [11].

Previous studies have shown that BPA could have various health effects, including diabetes [12], [13], coronary artery stenosis [14], [15], heart rate variability and blood pressure [16], abnormal liver function [17], childhood neurobehavioral problems [18], oxidative stress and inflammation [10], male sexual dysfunction [19], [20], decreased semen quality [21], [22], and adverse birth outcomes [23]. Especially in women, BPA has been associated with abnormal pubertal development [24], [25], externalizing behaviors [26], recurrent miscarriages [27], and premature delivery [28].

Studies have also reported a potential association between BPA exposure and asthma. Animal studies suggest that BPA might affect the development of asthma-related conditions by promoting allergic immune responses [29]–[33]. BPA has also been shown to promote eosinophilic bronchial inflammation and airway responsiveness in mice [9], [34] and production of airway secretary proteins, which is one of the hallmarks of asthma, in rhesus monkeys [35]. In humans, a relationship between prenatal urinary BPA and wheezing has been reported among children under the age of 3 years [36]. These findings are supported by a reported association between pre- and postnatal BPA exposure and wheezing and asthma development in preschool children [37].

However, to the best of our knowledge, there is little evidence regarding the effects of BPA exposure on asthma in school-age children. Furthermore, examining the association in Korea, where relatively low BPA concentration of school-age children (1.2 µg/L) compared to that of USA (2.7 µg/L) had been reported, gives extra information [38], [39]. In the present study, we hypothesized that exposure to BPA in school-age children is associated with asthma-related outcomes such as wheezing, asthma, and PC_20_, which is defined as the methacholine concentration that causes a decrease in FEV_1_ of 20% from baseline.

## Materials and Methods

### Study sample and data collection

**Figure 1:**
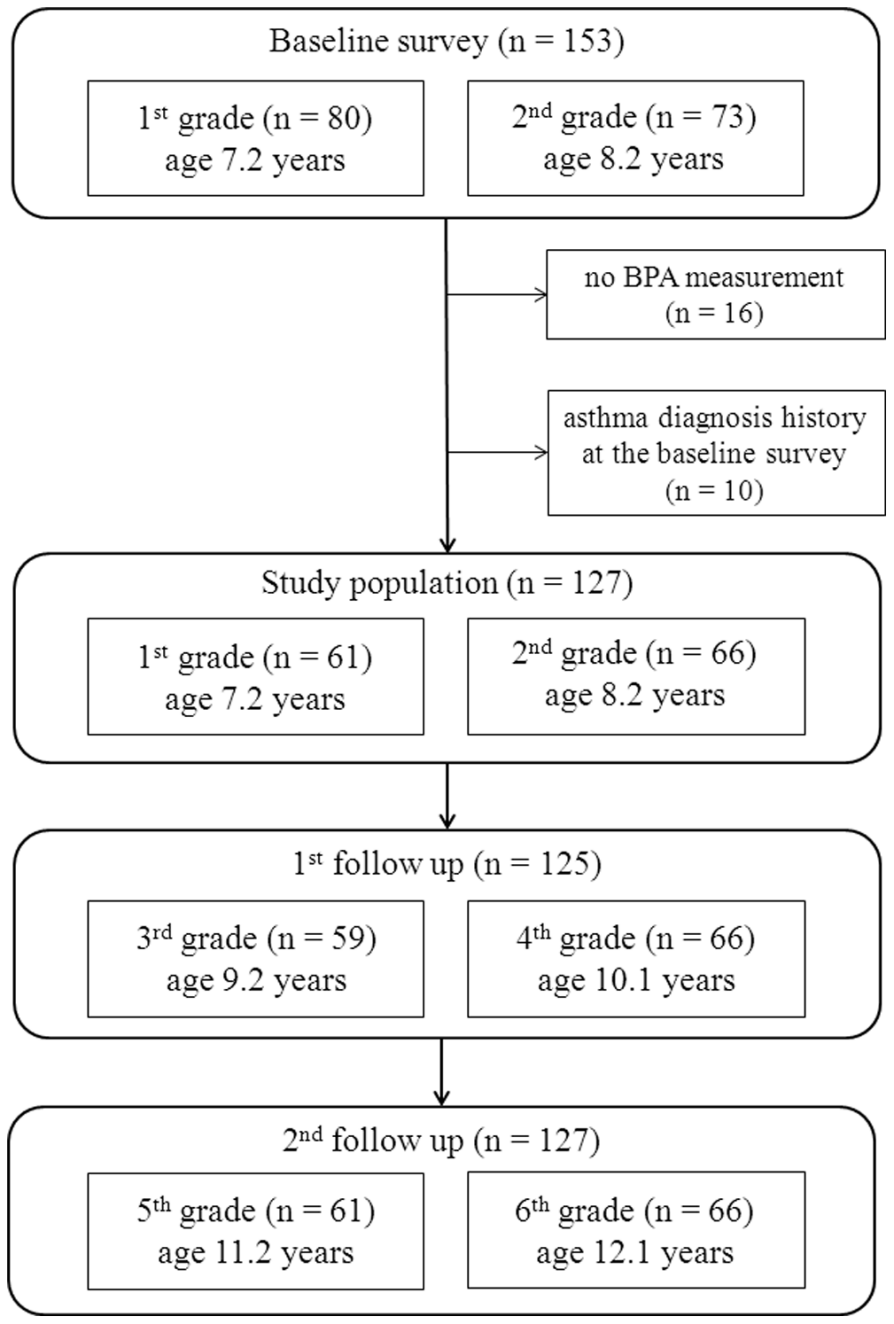
Overview of study population sampling and follow-up.

In 2005, all the 1^st^ grade (n = 92) and 2^nd^ grade children (n = 96) in an elementary school in Seoul, Korea, were invited to the study. Of a total of 188 children aged 7–8 years, parents of 153 schoolchildren agreed to enroll for the baseline survey, which consisted of a methacholine challenge test, urinary BPA measurement, and the International Study of Asthma and Allergies in Childhood (ISAAC) questionnaire, answered by the parents or guardian. Of the original sample, the participants without a BPA measurement (n = 16) or with a previous asthma diagnosis (n = 10) at baseline were subsequently excluded from the analysis, resulting in 127 children. In 2007, 125 of the 127 children who were then aged 9–10 years participated in the first follow-up survey, and, in 2009, all of the original 127 children who were then aged 11–12 years participated in the second follow-up survey (Figure 1). The follow-up surveys consisted of the ISAAC questionnaire and the methacholine challenge test.

Written informed consent was obtained from parents or guardians of all participating children, and the study protocols were reviewed and approved by the Institutional Review Board at the Dankook University Medical Center.

### Exposure assessment

All participating children were asked to fast for more than 8 h before the survey. Spot urine samples (50 mL) were collected between 0900 and 1200 h from participants at the baseline survey (n = 127) in conical tubes (SPL Lifesciences, Pocheon, Gyunggi-do, Korea). Urine samples were stored at −20°C in the freezer and sent to the laboratory (NeoDin Medical Institute, Seoul, Korea) within 90 min. Urine samples were buffered using 30 µL of 2M sodium acetate (pH 5.0), and a mixture of 10 µL ß-glucuronidase/sulfatase (Sigma, St. Louis, MO, USA) and 25 µL BPA (RING-13C12, 99%, Cambridge Isotope Lab, Inc., Tewksbury, MA, USA) was added. The samples were incubated at 37°C for 3 h to deconjugate the glucuronidated BPA, and 100 µL of 2N HCl was added after incubation. The extract was dehydrated with nitrogen gas and then reconstituted with 1 mL of HPLC-grade H_2_O in a 2 mL glass vial. Each batch of samples included a quality control sample and a blank. We added the quality control sample in pooled urine with a mixture of BPA standard. Liquid-liquid extraction was performed with Agilent Eclipse plus C18, 3.5 µm, 2.1×100 mm. The mobile phase was 60∶40 (v/v) acetonitrile:water, and the flow rate was 0.4 mL/min. Total BPA, including the free and conjugated forms, was measured using a high performance liquid chromatography-mass selective detector (HPLC-MS/MS, Agilent Triple Quad 6410, Santa Clara, CA, USA). The BPA concentrations in all the urinary samples were above the limit of detection (0.005 µg/L). The concentration of urinary BPA was adjusted for creatinine measured from the same urine sample to eliminate the influence caused by the different urinary excretion rates between participants [11].

### Outcome measures

Wheezing was defined using the ISAAC question “Has your child had wheezing or whistling in the chest in the last 12 months?”. Children were considered to have asthma (current asthma) when either of the following criteria was met: 1) wheezing or the use of asthma medication in the previous 12 months combined with a PC_20_≤8 mg/mL or 2) wheezing or the use of asthma medication in the previous 12 months combined with a history of an asthma diagnosis or a history of wheezing. Incident asthma was defined as the first detection of current asthma without having satisfied the criteria of current asthma at any previous survey.

The response to the methacholine challenge is expressed as PC_20_. To evaluate PC_20_, which is an indicator of airway hyperresponsiveness, participants inhaled increasing concentrations of methacholine (0.625, 1.25, 2.5, 5, 12.5, and 25 mg/dL) using a nebulizer until the FEV_1_ measured by a portable spirometer (Microspiro HI-298, Chest Corporation, Tokyo, Japan) decreased by at least 20% from the baseline value. The PC_20_ was calculated using a log-dose response curve [40].

### Covariates

The parental asthma history was reported on the baseline questionnaire. Fetal tobacco smoke exposure was defined as active maternal smoking or the report of a smoker in the home during pregnancy. Environmental tobacco smoke exposure was defined as active maternal smoking during the first year after delivery, maternal smoking at the time of the survey, or the report of a smoker in the home from delivery until the time of the survey. Pet ownership was defined as having ever kept dogs or cats as pets from delivery until the time of the surveys.

### Statistical analyses

Urinary BPA concentration was adjusted for creatinine measured from the same urine sample (i.e., urinary BPA concentration divided by urinary creatinine concentration) and log-transformed to approximate a normal distribution. The associations between urinary BPA concentration at 7–8 years of age and the dichotomous outcome variable, e.g., wheezing and current asthma, were analyzed using generalized estimating equations (GEE) with a logit link. A marginal Cox model with a robust sandwich estimator of variance was applied to analyze the association between BPA exposure and risk of incident asthma considering the grade-at-enrollment dependence due to the possibility of clustering within the grade. The proportional hazards assumption was tested using a time-dependent explanatory variable. For those with incident asthma, the time at risk was considered as the number of years from the baseline survey to the mid-point between the previous survey and the survey when the incident asthma was observed and, for those without incident asthma, as the number of years between the baseline survey and the last follow-up survey. Due to the difficulty of assigning the exact time at risk and the relatively long period between the surveys, the association between BPA concentration and incident asthma was also analyzed using a logistic regression model.

The associations between urinary BPA concentration at 7–8 years of age and PC_20_ at the 3 time points (7–8, 9–10, and 11–12 years of age) were analyzed in two steps. First, a generalized additive mixed model was constructed to graphically examine the linearity of the association. Then, a linear mixed model using repeated-measures analysis and a random effect of grade-at-enrollment was constructed to analyze the relationship between BPA concentration at 7–8 years of age and PC_20_ at 3 time points. Logistic and linear regression models were used to analyze the association between BPA concentration and wheezing, current asthma, and PC_20_ at each time point.

The child’s sex, parental asthma history, fetal tobacco smoke exposure, and pet ownership were selected as covariates based on earlier literature reviews [36], [37]. Potential confounders, such as environmental tobacco smoke exposure, history of breastfeeding, cockroach sensitization, and maternal education level were evaluated using bivariate analyses. Covariates that predicted wheezing at *P*≤. 20 in the bivariate analyses were added to the initial multivariable GEE model and retained if the estimate of the association between BPA at 7–8 years of age and wheezing changed >10%, which resulted in the child’s sex, parental asthma history, fetal tobacco smoke exposure, environmental tobacco smoke exposure, and pet ownership as the covariates in the analysis. The GEE with a logit link, logistic regression, and linear regression models were also adjusted for grade at enrollment.

In the secondary analyses, potential interaction was tested by adding cross product term between BPA concentration and each covariate in the main analysis. The participants were stratified into subgroups based on sex and analyzed using the marginal Cox model for clustered data and a linear mixed model.

SAS version 9.3 (SAS Institute Inc., Cary, NC, USA) was used for statistical analyses, and R version 2.14.2 (The Comprehensive R Archive Network: http://cran.r-project.org) was used for visualization. Two-sided *P* values <.05 were used to indicate statistical significance.

## Results

Of the 127 participants, 54.3% were male, 4.7% had a parental asthma history, 17.3% experienced fetal tobacco smoke exposure, and 26.8% experienced environmental tobacco smoke exposure. Difference between baseline characteristics of children who had current asthma during the study period and those of children who did not was not observed (Table 1). When comparing the children that were included in the analysis with those excluded, there were no differences except for a slightly higher level of cockroach sensitization in the children that were included in the analysis (Table S1 in File S1).

**Table 1:** Baseline characteristics of the study participants stratified by children who had current asthma during the study period and children who did not [*n* (%)].

Characteristic	Children with asthma (*n* = 18)	Children without asthma (*n* = 109)	*P* value*
Sex			.53
Boy	11 (61.1)	58 (53.2)	
Girl	7 (38.9)	51 (46.8)	
Parental asthma history			.09
No	14 (77.8)	99 (90.8)	
Yes	1 (5.6)	5 (4.6)	
Missing	3 (16.7)	5 (4.6)	
Fetal tobacco smoke exposure†			.94
No	15 (83.3)	90 (82.6)	
Yes	22 (16.7)	19 (17.4)	
Environmental tobacco smoke exposure§			.50
No	12 (66.7)	81 (74.3)	
Yes	6 (33.3)	28 (25.7)	
Pet ownership‡			.45
No	15 (83.3)	82 (75.2)	
Yes	3 (16.7)	27 (24.8)	
Breast-fed			.11
No	5 (27.8)	35 (32.1)	
<3 months	4 (22.2)	18 (16.5)	
3–5 months	0 (0)	16 (14.7)	
≥6months	4 (22.2)	30 (27.5)	
Did not answer	5 (27.8)	10 (9.2)	
Cockroach sensitization			.67
No	17 (94.4)	103 (94.5)	
Yes	0 (0)	3 (2.8)	
Did not answer	1 (5.6)	3 (2.8)	
Maternal education			.67
< High school	3 (11.9)	13 (11.9)	
High school	7 (38.9)	51 (46.8)	
>High school	5 (27.8)	34 (31.2)	
Did not answer	3 (16.7)	11 (10.1)	
Paternal education			.19
< High school	3 (16.7)	6 (5.5)	
High school	6 (33.3)	50 (45.9)	
>High school	6 (33.3)	43 (33.3)	
Did not answer	3 (16.7)	3 (9.2)	

* *P* value was estimated based on Chi-square test or Fisher’s exact test.

† Active maternal smoking during pregnancy or presence of a smoker in the home during pregnancy.

‡ Active maternal smoking during the first year after delivery, current maternal smoking, or presence of a smoker in the home after delivery until the present time.

§ Having had a pet dog or cat after delivery until the present time.

Size of the wheal produced by the cockroach antigen ≥3 mm and larger than size of the wheal produced by histamine.

The geometric mean of urinary BPA concentration was 1.02 µg/L, 1^st^ quartile 0.63 µg/L, median 0.97 µg/L, 3^rd^ quartile 1.67 µg/L, and maximum 21.37 µg/L. The distribution of urinary BPA concentrations was positively skewed. In the present study, 9 children were assessed to have current asthma only at 9–10 years, 7 children only at 11–12 years, while 2 children both at 9–10 years and 11–12 years of age. The one-unit increase in log-transformed, creatinine-adjusted urinary BPA concentration measured at 7–8 years of age was associated with wheezing (odds ratio [OR], 2.48; 95% confidence interval [CI], 1.15–5.31; *P* = .02) and current asthma (OR, 2.35; 95% CI, 1.03–5.32; *P* = .04) at ages up to 11–12 years. A relationship between urinary BPA concentration and the risk of incident asthma was also observed (hazard ratio [HR], 2.13; 95% CI, 1.51–3.00; *P*<.001; Table 2). A statistically significant association was also observed between BPA concentration and incident asthma in the logistic regression model (OR, 2.44; 95% CI, 1.11–5.36; *P* = .03).

**Table 2:** Association of urinary BPA concentrations (log transformed, µg/g creatinine) at 7–8 years with wheezing and asthma over 11–12 years of age, by longitudinal analyses.

Outcome	No.*	OR† or HR‡ (95% CI)	*P* value
Wheeze	28/335	2.48 (1.15–5.31) †	.02
Current Asthma	20/252	2.35 (1.03–5.32) †	.04
Incident Asthma	18/127	2.13 (1.51–3.00) ‡	<.001

HR, hazard ratio.

* Number with outcome/total number for analysis.

† Generalized estimating equation with a logit link model adjusted for gender, parental asthma history, fetal and environmental tobacco smoke exposure, pet ownership, and grade at enrollment.

‡ Marginal Cox model considering grade-at-enrollment clustering adjusted for gender, parental asthma history, fetal and environmental tobacco smoke exposure, and pet ownership.

**Figure 2:**
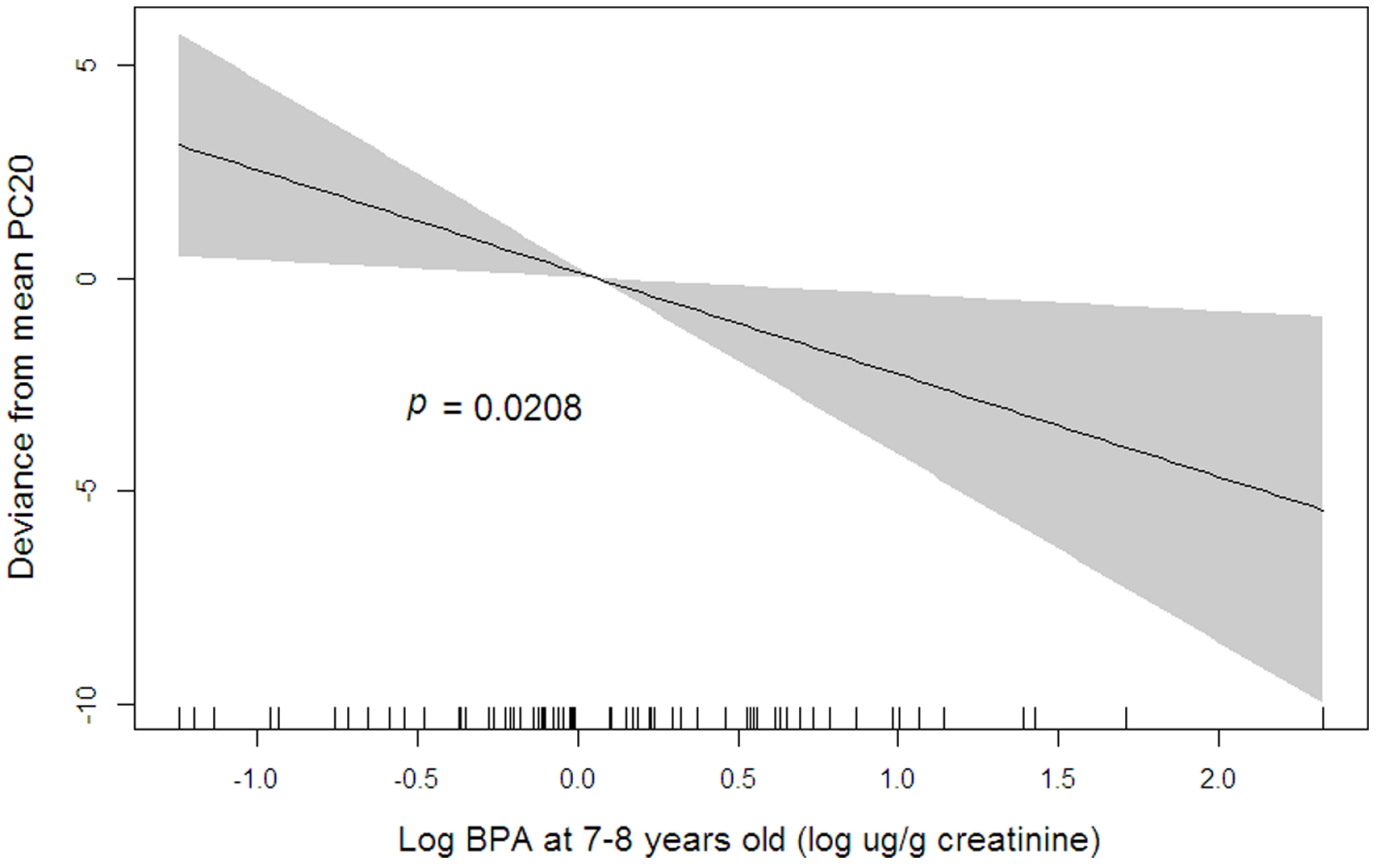
Relationship between urinary BPA concentration and PC_20_. Penalized regression spline of log-transformed urinary BPA concentrations at 7–8 years on PC_20_ at ages up to 11–12 years. Solid lines, spline curve; shaded area, 95% confidence intervals. The model is adjusted for gender, parental asthma history, fetal and environmental tobacco smoke exposure, and pet ownership.

The penalized regression spline showed an almost linear association between BPA at 7–8 years of age and PC_20_ at ages up to 11–12 years (Figure 2). In the linear mixed model, the association between BPA at 7–8 years of age and PC_20_ at ages up to 11–12 years was significant (ß = −2.33; *P* = .02).

The analysis of the associations between BPA at 7–8 years of age and wheezing, PC_20_, and current asthma at each time point resulted in significant relationships with wheezing, PC_20_, and current asthma at 9–10 years of age only (Table 3). When the interaction term between BPA and each covariate was added to the multivariable model, significant interactions were not found. When stratified by sex, a significant association between BPA and incident asthma (HR, 2.45; 95% CI, 2.18–2.76; *P*<.001) and a marginally significant association between BPA and PC_20_ (ß = −2.59, *P* = .09; Table S2 in File S1) were observed only in girls.

**Table 3:** Association of urinary BPA concentrations (log transformed, µg BPA/g creatinine) at 7–8 years with wheezing, PC_20_, and current asthma at 7–8, 9–10, and 11–12 years of age.

	Log BPA (µg/g creatinine) at 7–8 y								
	Wheeze*			PC_20_ †			Current asthma*		
Age (years)	No.‡	OR (95%CI)	*P* value	No.§	ß (SE)	*P* value	No.‡	OR (95%CI)	*P* value
7–8	8/120	3.08 (0.86–11.09)	.09	64	−2.01 (1.22)	.11	NA		
9–10	11/121	4.24 (1.35–13.28)	.01	19	−6.37 (2.26)	.02	11/125	3.64 (1.23–10.76)	.02
11–12	9/122	1.80 (0.69–4.71)	.23	14	−3.64 (2.50)	.20	9/127	1.87 (0.72–4.86)	.20

*Logistic regression model adjusted for gender, parental asthma history, fetal and environmental tobacco smoke exposure, pet ownership, and grade at enrollment.

† Linear regression model adjusted for gender, parental asthma history, fetal and environmental tobacco smoke exposure, pet ownership, and grade at enrollment.

‡ Number with outcome/total number for analysis.

§ Total number for analysis.

For the sensitivity analysis, we conducted the analyses after including the children who had been diagnosed with asthma before the baseline survey (n = 10). This did not change the result substantially (Table S3 in File S1). When excluding the children who satisfied the criteria of current asthma at the baseline survey, BPA concentration at 7–8 years of age was significantly associated with increased risk of incident asthma (HR, 1.64; 95% CI, 1.10–2.45; *P* = .02). Significant association of BPA with PC_20_ at 9–10 years and marginally significant association with wheezing at 9–10 years of age was also observed. The trend of association between BPA and current asthma was similar although attenuated, partly due to small sample size (Table S4 in File S1).

## Discussion

In the present study, we found associations between the urinary BPA concentration in the earlier years of children in elementary school and wheezing, asthma, and PC_20_ in the later years. A potential modifying effect of sex on these associations was also observed.

Previous studies have reported that prenatal or postnatal exposure to BPA is associated with an increased risk of wheezing and asthma. One birth cohort study of 398 mother-child pairs demonstrated that prenatal urinary BPA concentrations above, versus below, the median are associated with the child’s wheezing at 6 months (OR, 2.27; 95% CI, 1.28–4.06) but not at older ages until 3 years of age [36]. Another birth cohort study of 568 mother-child pairs showed that urinary BPA concentrations at 3 years are associated with wheezing at 5 and 6 years of age, and urinary BPA concentrations at 7 years are associated with wheezing at 7 years of age. A one-unit increase in log-transformed, creatinine-adjusted urinary BPA concentrations at 3, 5, and 7 years is associated with asthma at a single assessment by a physician between 5 and 12 years of age (OR, 1.5; 95% CI, 1.1–2.0 for BPA at 3 years; OR, 1.4; 95% CI, 1.0–1.9 for BPA at 5 years; OR, 1.5; 95% CI, 1.0–2.1 for BPA at 7 years of age) [37]. Our results are similar to the previous findings, although the effect size is relatively large in the current study, which may be explained by the differences in outcome definition, study population, and adjusting covariates.

In the current study, urinary BPA at 7–8 years was associated with asthma-related outcomes such as wheezing, asthma, and PC_20_ at 9–10 years, but not at 7–8 years or 11–12 years of age. Due to the lack of mechanistic studies investigating the time lag between BPA exposure and occurrence of asthma-related outcomes, we have no definite explanation for this finding. Pubertal stage may play a role; it has been reported that pubertal stage is related to the development and progression of asthma [41]–[44]. Alternatively, observed null association with current asthma at 11–12 years of age might reflect the fact that only a small number of incident asthma cases occur in later years in elementary school, and the incidence of wheezing and asthma continues to decline from childhood to adolescence or greater non-differential misclassification due to longer time interval [45]. Further research considering the pubertal stage is warranted.

The mechanism behind the present findings is still unclear; however, the oxidative stress pathway with BPA exposure could be suggested as a possible explanation. BPA is known to cause oxidative stress in rats [46]–[49] and humans [10], and growing evidence indicates that oxidative stress and subsequent mitochondrial dysfunction are associated with BPA-induced damage [50], [51]. Increased production of reactive oxygen species and decreased anti-inflammatory capacity could enhance the susceptibility to the insults, such as air pollution, which results in chronic airway inflammation and asthma [52], [53].

The results of the present study also suggest that the effects of BPA on asthma-related outcomes might be mediated, at least in part, by an endocrine-disrupting mechanism. It was reported that the prevalence of asthma is higher in boys before puberty and in girls and women after puberty [54]. Changes in hormonal status, such as with estrogen, have been suggested as one possible factor causing this phenomenon [45], [55]. Estrogen has been demonstrated to encourage T-helper 2 (T_H_2) polarization, class switching of B cells to the production of immunoglobulin E, and the degranulation of mast cells [56]–[59]. In epidemiologic studies, early menarche is associated with a higher prevalence of asthma in adult women [60], and the diagnosis of asthma increases in postmenopausal women who receive hormone replacement therapy [61]. Meanwhile, BPA acts imperfectly as estrogen in numerous organs [62], and female mice that were exposed to BPA prenatally demonstrate increased airway and lung inflammation, whereas the male mice exposed to BPA prenatally did not [63]. The results of these studies and ours suggest that BPA affects asthma-related outcomes by disrupting the endocrine system. However, the mechanisms underlying the sex-specific effect of and susceptibility to BPA are not yet fully understood [29], [55], [64].

BPA concentration in the present study was markedly lower than the previously reported BPA concentration in the United States [11], [38]. Geographic variance of the BPA concentration has been reported not only in the children [38], [39] but also in different age group population [11], [17], [65], [66]. The observed lower BPA concentration in the present study might also be attributable, in part, to the study design using overnight fasting spot urine samples [67], [68]. However, it has been reported that urinary BPA concentration did not decrease rapidly with fasting time [69], and another study following five fasting individual’s spot urine BPA concentration has also demonstrated the decline of BPA concentration with gentle slopes during the first 24 h and fluctuated at lower levels during the next 24 h [70]. This might be due to accumulation of BPA in body tissue, such as fat or exposure to nonfood source and could lead to reduction in the variability of BPA concentration and potential misclassification [71]. Due to these traits, fasting urinary BPA concentration has been used as an indicator of exposure in the previous studies [16], [72]. Further, the random variation in BPA concentrations may have shifted the association toward the null.

Exposure to BPA is thought to be mainly from dietary route [67] and the observed associations could be confounded by the chemicals that were taken with BPA. For instance, mercury and BPA share common exposure source such as canned tuna fish [73], [74]. Although exposure to mercury has been associated with immunotoxic effects [75]–[78], we could not find previous literature supporting the association between exposure to mercury and asthma. Further study exploring the co-exposure of BPA and other environmental risk factors including phthalate, other phenolic compounds, heavy metals, and persistent organic compounds is warranted to assess potential effect modification or confounding [79].

The present study has some strengths. First, the longitudinal study design addressed the potential of temporal ambiguity and recall bias. Second, the use of the objective methacholine challenge test provided a more accurate diagnosis and reduced the potential of misclassification. Furthermore, the reliability of the results was demonstrated by the consistency of results between the parent-reported presence of wheezing and asthma and the results from the objective methacholine challenge test.

The major limitation in the current study is the small sample size. In addition, the selection of participants from one elementary school may have resulted in drawbacks in generalizability. Future studies should include a more representative sample of sufficient size to confirm the findings of the present study. Although PC_20_ is a commonly used indicator for airway hyperresponsiveness, its use is limited in participants whose decrease in FEV_1_ is less than the cut-off value before the maximal concentration is reached [80], which happens with the majority of the participants in epidemiologic studies [81]. However, despite the weakness regarding censoring, previous studies have reported that PC_20_ correlates well with other indicators of airway hyperresponsiveness [82]–[84] and can be used as a reliable indicator of airway hyperresponsiveness [85], [86]. Lastly, due to the lack of a BPA measurement before 7–8 years of age, the possibility that the observed association is due to earlier exposure and that the BPA exposure at 7–8 years of age is reflective of this could not be assessed. Further studies are required to confirm the temporal specifics regarding vulnerability.

We found that the urinary BPA concentration at 7–8 years was associated with wheezing, asthma, and PC_20_ at ages up to 11–12 years. These findings provide information about the health effects of BPA exposure in school-age children and support public health initiatives to protect the health of a susceptible population such as children.

## Supporting Information

File S1 **This file contains Table S1, Table S2, Table S3, and Table S4. Table S1**, Baseline characteristics of the study population included and excluded in the current study. **Table S2**, Association of urinary BPA concentrations (log transformed, µg BPA/g creatinine) at 7–8 years with incident asthma and PC20 over 11–12 years of age stratified by gender. **Table S3**, Association of urinary BPA concentrations (log transformed, µg BPA/g creatinine) at 7–8 years with wheezing, PC20, and asthma at 7–8, 9–10, and 11–12 years of age, including the children who had been diagnosed with asthma before 7–8 years of age. **Table S4**, Association of urinary BPA concentrations (log transformed, µg BPA/g creatinine) at 7–8 years with wheezing, PC20, and current asthma at 7–8, 9–10, and 11–12 years of age, excluding the children who satisfied the criteria of current asthma at the baseline survey.
(PDF)

File S2 **Data set.**
(CSV)
