# Supplementary material for: Bisphenol A Exposure and Asthma Development in School-Age Children: A Longitudinal Study
Source: PLoS One. 2014 Oct 30;9(10):e111383. doi: 10.1371/journal.pone.0111383 (PMC4214730; doi:10.1371/journal.pone.0111383)
Supplement: File S1 — This file contains Table S1, Table S2, Table S3, and Table S4. Table S1, Baseline characteristics of the study population included and excluded in the current study. Table S2, Association of urinary BPA concentrations (log transformed, µg BPA/g creatinine) at 7–8 years with incident asthma and PC20 over 11–12 years of age stratified by gender. Table S3, Association of urinary BPA concentrations (log transformed, µg BPA/g creatinine) at 7–8 years with wheezing, PC20, and asthma at 7–8, 9–10, and 11–12 years of age, including the children who had been diagnosed with asthma before 7–8 years of age. Table S4, Association of urinary BPA concentrations (log transformed, µg BPA/g creatinine) at 7–8 years with wheezing, PC20, and current asthma at 7–8, 9–10, and 11–12 years of age, excluding the children who satisfied the criteria of current asthma at the baseline survey. (PDF) [file pone.0111383.s001.pdf]

Table S1. Baseline characteristics of the study population included and excluded in the current study

Characteristic	Included (*n*=127)	Excluded (*n*=26)	*P* value*
Male, no. (%)	69 (54.3)	12 (46.2)	.45
Parental asthma history, no. (%)	6 (4.7)	0 (0)	.52
Fetal tobacco smoke exposure, no. (%)†	22 (17.3)	4 (15.4)	.81
Environmental tobacco smoke exposure, no. (%)‡	34 (26.8)	7 (26.9)	.99
Pet ownership, no. (%)§	30 (23.6)	4 (15.4)	.36
Breast-fed, no. (%)	73 (57.5)	14 (53.9)	.38
Cockroach sensitization, no. (%) ‖	3 (2.4)	0 (0)	.01
Maternal education > 12 years, no. (%)	39 (30.7)	9 (34.6)	.85
Age, mean± SD	7.7 ± 0.6	7.5 ± 0.6	.11

**P* value were based on Chi-square test and Fisher's exact test for categorical variables, and t-test for continuous variables.

†Active maternal smoking during pregnancy or presence of a smoker in the home during pregnancy.

‡Active maternal smoking during the first year after delivery, current maternal smoking, or presence of a smoker in the home after delivery until the present time.

§Having had a pet dog or cat after delivery until the present time.

‖ Size of the wheal produced by the cockroach antigen ≥ 3mm and larger than size of the wheal produced by histamine.

Table S2. Association of urinary BPA concentrations (log transformed, µg BPA/g creatinine) at 7-8 years with incident asthma and PC_{20} over 11-12 years of age stratified by gender

	Asthma		PC_{20}	
	HR (95% CI)*	*P* value	ß (SE)†	*P* value
Overall	2.13 (1.51-3.00)	<.001	-2.33 (0.98)	0.02
Male	2.25 (0.67-7.55)	0.19	-2.78 (1.69)	0.11
Female	2.45 (2.18-2.76)	<.001	-2.59 (1.45)	0.09

HR, hazard ratio.

*Marginal Cox model considering grade-at-enrollment clustering adjusted for gender, parental asthma history, fetal and environmental tobacco smoke exposure, and pet ownership.

† Linear mixed model with a random effect of grade-at-enrollment clustering adjusted for gender, parental asthma history, fetal and environmental tobacco smoke exposure, and pet ownership.

Table S3. Association of urinary BPA concentrations (log transformed, μg BPA/g creatinine) at 7-8 years with wheezing, PC_{20}, and asthma at 7-8, 9-10, and 11-12 years of age, including the children who had been diagnosed with asthma before 7–8 years of age

	Wheeze*			PC_{20}†		
Age (years)	No.‡	OR (95%CI)	*P* value	No.‡	ß (SE)	*P* value
7–8	13/130	1.38 (0.53–3.62)	.52	71	-2.05 (1.23)	.10
9–10	12/131	3.48 (1.20–10.09)	.02	22	-6.19 (2.40)	.02
11–12	10/132	1.71 (0.67–4.34)	.26	15	-2.92 (3.46)	.43

*Logistic regression model adjusted for gender, parental asthma history, fetal and environmental tobacco smoke exposure, pet ownership, and grade at enrollment.

†Linear regression model adjusted for gender, parental asthma history, fetal and environmental tobacco smoke exposure, pet ownership, and grade at enrollment.

‡Number with outcome/total number for analysis.

Table S4. Association of urinary BPA concentrations (log transformed, μg BPA/g creatinine) at 7-8 years with wheezing, PC_{20}, and current asthma at 7-8, 9-10, and 11-12 years of age, excluding the children who satisfied the criteria of current asthma at the baseline survey

	Log BPA (μg /g creatinine) at 7-8 y								
	Wheeze*			PC_{20}†			Current asthma*		
Age (years)	No.‡	OR (95%CI)	*P* value	No.§	ß (SE)	*P* value	No.‡	OR (95%CI)	*P* value
7–8	0/112			60	-2.03 (1.28)	.12	NA		
9–10	9/113	3.12 (0.84–11.60)	.09	18	-7.01 (2.25)	.01	9/117	2.54 (0.75–8.58)	.13
11–12	8/114	1.71 (0.56–5.22)	.34	13	-3.58 (2.54)	.23	8/119	1.79 (0.59–5.45)	.31

*Logistic regression model adjusted for gender, parental asthma history, fetal and environmental tobacco smoke exposure, pet ownership, and grade at enrollment.

†Linear regression model adjusted for gender, parental asthma history, fetal and environmental tobacco smoke exposure, pet ownership, and grade at enrollment.

‡Number with outcome/total number for analysis.

§Total number for analysis
